# Consumer-Based Sensory Characterization of Steviol Glycosides (Rebaudioside A, D, and M)

**DOI:** 10.3390/foods9081026

**Published:** 2020-07-31

**Authors:** Ran Tao, Sungeun Cho

**Affiliations:** 1 Department of Food Science & Human Nutrition, Michigan State University, East Lansing, MI 48824, USA; taoran3@msu.edu; 2 Department of Poultry Science, Auburn University, Auburn, AL 36849, USA

**Keywords:** stevia, steviol glycosides, rebaudiosides, Reb A, Reb D, Reb M, aftertaste, CATA, PROP

## Abstract

Rebaudioside (Reb) D and M are the recent focus of the food industry to address the bitter taste challenge of Reb A, which is the most commonly used steviol glycoside in natural sweetener stevia. This study evaluated the sensory characteristics of Reb A, D, and M, compared to 14% (*w*/*v*) sucrose, using a consumer panel and explored the relationship between 6-n-Propylthiouracil (PROP) taster status (i.e., non-tasters, medium tasters, supertasters) and the perceived intensity of sweet and bitter tastes of the three steviol glycosides. A total of 126 participants evaluated the intensities of in-mouth, immediate (5 s after expectorating), and lingering (1 min after expectorating) sweetness and bitterness of 0.1% Reb A, D, M, and 14% sucrose and described the aftertaste of the sweeteners by using a check-all-that-apply (CATA) question. The results showed that in-mouth sweetness and bitterness of Reb D and M were not significantly different from sucrose, unlike Reb A which showed significant bitterness. However, Reb D and M showed more intense lingering sweetness than sucrose. The CATA analysis resulted that Reb D and M were closer to positive attribute terms and also to sucrose than Reb A, but Reb D and M were still considered artificial, which may cause them to be perceived negatively. When comparing among PROP taster groups, no significant differences in the perceived sweetness and bitterness of the three steviol glycosides were found. This study generates important information about Reb A, D, and M for the food industry, especially working with products formulated to deliver reductions in sugar using a natural high-intensity sweetener, stevia.

## 1. Introduction

Artificial sweeteners are widely used in a variety of foods and beverages as a sugar substitute that mimics the effect of sugar on taste without adding calories. However, consumers have a negative perception of artificial sweeteners not only due to aversive sensations such as bitter off-taste [1,2] but also due to potential health risks and demand more natural options [3]. To respond to the consumers’ demand for natural sugar substitutes with low/zero calories, the food industry has focused on stevia, which is a natural high-intensity non-nutritive sweetener. Stevia (*Stevia Rebaudiana* Bertoni) is a shrub native to Paraguay, and the leaves of stevia have been used to sweeten teas for hundreds of years in Paraguay and Brazil [4,5]. Stevia is the source of many different types of steviol glycosides, which are the sweetening compounds in stevia leaves [6]. Stevioside and rebaudioside (Reb) A are the major sweet compounds among the steviol glycosides [6] and are the most widely used steviol glycosides on the market according to a Mintel Global New Products Database (GNPD) product search [7]. However, stevioside and Reb A exhibit bitter and licorice off-taste [8,9,10,11,12,13], which pose challenges to product formulation.

To overcome the taste challenges of stevioside and Reb A, the researchers and food industry have looked into other minor steviol glycosides in the stevia leaves to provide better sugar reduction solutions. Several studies have reported that the two minor steviol glycosides, Reb D and M, elicit significantly less bitterness with better sweetness than Reb A and also work well in products without sacrificing the taste [14,15,16,17,18,19]. Prakash et al. [16] reported that Reb M had less bitterness and astringency than Reb A. Most of the studies investigating sensory characteristics of steviol glycosides were conducted within a specific range of 5–10% sweetness equivalency related to sucrose (SE) [8,10,11,13,16]. Little research was done at high concentrations for high-sugar applications such as frozen desserts, which generally contain 13–22% sucrose *w*/*v* [20], although sweetness potency of stevia heavily depends on the SE [13].

For sensory characterization of food products, sensory descriptive analysis using trained assessors is the most widely used method, but it is time-consuming to train a panel. Less time consuming and more flexible methodologies such as check-all-that-apply (CATA) or intensity scales using consumers have been discussed in the last two decades [21]. It has been reported that consumers were capable of evaluating sensory attributes of various products, showing good agreement between consumers and trained assessors in terms of discrimination, reproducibility, and consensus [22,23,24,25]. Although Worch et al. [24] found that the trained panel showed greater consensus among each other, the larger sample size of consumers compensated for the higher variability. Moskowitz [26] suggested that a minimum of 40–50 people was needed to get stable averages, and the averages would not be affected by the base size much once the participant number exceeded 80. Ares et al. [27] also indicated that 80 consumers would be sufficient to get stable results when samples had large differences, but caution would be needed if samples had smaller differences or more complex attributes.

CATA is also often used to determine the characteristics of a product from a consumer perspective, which allows the consumers to describe a product by selecting terms from a given list that would match the product [28]. CATA questions have been used for a variety of foods and beverages [28,29,30,31,32], and these studies showed that CATA was a simple way to understand consumer perception on the sensory profile of a product.

Phenylthiocarbamide (PTC) and 6-n-Propylthiouracil (PROP) are bitter-tasting compounds that have been used to test people’s sensitivity to bitter taste. Supertasters are a group of people who perceived intense bitter taste from PTC and PROP, while non-tasters barely detect the bitterness of them [33]. It has been reported that individuals have different sensitivity to the aftertaste of high-intensity sweeteners [34], and thus researchers have long been interested in understanding the relationship between PROP status (e.g., non-tasters vs. supertasters) and perceived taste intensities of high-intensity sweeteners. Bartoshuk [35] and Drewnowski et al. [36] found a significant difference between non-tasters and supertasters in the bitterness of saccharin at low concentrations. Zhao and Tepper [37] also suggested that supertasters perceived more bitterness and sweetness than non-tasters in carbonated soft drinks with artificial sweeteners, including sucralose, aspartame, acesulfame-K. However, Horne et al. [38] did not find a relationship between PROP taster status and the sweetness and bitterness of saccharin and acesulfame-K. Rankin et al. [39] failed to find any significant difference in bitterness between supertasters and non-tasters in cola drink sweetened with artificial sweeteners either. Risso et al. [40] looked into the effect of genetic variations on stevioside and found that the bitter taste receptor for PROP did not predict the bitterness perception of stevioside. However, little research was done to investigate the influence of PROP status on the perceived sweet and bitter taste intensities of novel steviol glycosides such as Reb D and M.

The primary objective of this study was to determine sensory characteristics of Reb A, D, and M, compared to 14% (*w*/*v*) sucrose, using a consumer panel. A secondary objective was to determine if there is a relationship between PROP taster status and the perceived intensities of the three steviol glycosides.

## 2. Materials and Methods

### 2.1. Materials

Sweeteners used in the study were 95% Reb A (ENLITEN^®^ 30000015 High Intensity Sweetener, Ingredion, Westchester, IL), 95% Reb D (BESTEVIA^®^ Reb D stevia leaf sweetener, Ingredion, Westchester, IL), 95% Reb M (BESTEVIA^®^ Reb M stevia leaf sweetener, Ingredion, Westchester, IL, USA), and sucrose (Smidge & Spoon^TM^, Kroger, Cincinnati, OH, USA). PROP (6-Propyl-2-thiouracil, #P3755, Sigma-Aldrich, St. Louis, MO), NaCl (Sigma-Aldrich, St. Louis, MO, USA), and filter papers (1.5 dia. cm, VWR Scientific Products, West Chester, PA, USA) were used to make paper disks for supertaster screening.

### 2.2. PROP Status Determination

The paper disks for PROP status determination were prepared following the method described by Zhao et al. [41]. Blank, NaCl, and PROP disks were prepared. Blank disks were used as the control. NaCl disks were made by placing filter papers in 1.0 mol/L NaCl solution for 30 s at room temperature and oven-dried for 1 h at 121 °C (250 °F). 50-mmol/L PROP solution at boiling temperature was used for PROP disks.

PROP testing and classification were based on Zhao et al. [41] and Zhao and Tepper [37]. Michigan State University SONA Paid Research Pool (https://msucas-paid.sona-systems.com) was used to recruit participants with age between 18 and 55. Participants were instructed to rinse their mouth with water, taste the paper disk for 15 s or until the disk is wet, discard the paper disk, and then rate the perceived intensity of the taste on the labeled magnitude scale (LMS). The participants would taste a blank, a NaCl, and a PROP disk in order with a 30 s break in between samples to minimize fatigue and carryover. The set was repeated after a 5 min break.

The LMS is a 100 mm quasi-logarithmic spacing vertical scale with verbal labels from “barely detectable” to “strongest imaginable” [42]. The scale set up was “no sensation” = 0, “barely detectable” = 1.5, “weak” = 6, “moderate” = 17, “strong” = 35, “very strong” = 52, and “strongest imaginable” = 100 [43,44]. The PROP score of participants was calculated based on the mean of the two replicates. Because the LMS is not equal in spacing, the difference between two scores when both ratings are at the higher end is less than when ratings are at the lower end. If the difference between two ratings was bigger than 30 mm, or bigger than 40 mm when both ratings were higher than “very strong”, the participant would be considered having bad reproducibility and would not be invited to the following water solution testing. Out of 224 participants, 27 were excluded.

Initially, “moderate” or below (≤17 mm on the LMS) and “very strong” or above (≥52 mm on the LMS) of PROP score were used to group participants into non-tasters and supertasters. The group means and 95% confidence interval were then calculated to set new cut-off scores. The new cut-off score for non-tasters was 10.3 and for supertasters was 70.7. Participants with scores in between were classified as medium tasters. When the PROP score of a participant was borderline, the NaCl score was used to help classify the person [41]. A participant would be classified as a non-taster if the person gave a non-taster borderline score and rated NaCl much higher than PROP (~30 mm difference on the LMS). When a participant was at the supertaster borderline and gave a much lower NaCl score than PROP, the person would be classified as a supertaster. Out of 197 remaining participants, 25 were identified as non-tasters and 55 were supertasters.

### 2.3. Subjects Demographics

Following the PROP test, participants were asked to provide some basic demographic information, including age, gender, ethnicity, educational level, weight, height, health condition, consumption frequency of low/zero sugar added products, consumption of sweeteners on a regular basis (at least once a month), and familiarity with stevia.

### 2.4. Consumer Testing

#### 2.4.1. Samples and Sample Preparation

All solutions were prepared using deionized water and the concentration of the sample is expressed in g/L (*w*/*v*). Sucrose at 14% was chosen as the control. Reb A, D, and M at 0.09% were used in a preliminary test (*n* = 31) to determine the relative sweetness to 14% sucrose. The result showed that 0.09% Reb M were not statistically different from 14% sucrose in sweetness intensity (*p* = 0.16), but there was still a 1.1 difference on a marked 15-cm line scale with descriptors of “not at all” and “extremely” as endpoint anchors. Another preliminary test (*n* = 65) was then conducted to prove the sweetness equivalency of Reb M to sucrose, using 0.09% and 0.12% Reb M and 10% and 14% sucrose. The result indicated that both 0.09% and 0.12% Reb M were not significantly different from 14% sucrose (*p* = 0.34 and *p* = 0.11, respectively), with 0.09% Reb M closer to 14% sucrose at 0.5 difference on a 15-cm line scale, comparing to a 1.0 difference between 0.12% Reb M and 14% sucrose. Since 0.09% Reb M was again lower in intensity on the scale, Reb M at 0.10% was chosen for the consumer testing. Reb A and D at 0.10% were used to compare the sensory characteristics of the three steviol glycosides at the same concentration. Thus, samples used for the testing were Reb A, D, and M at 0.10%, and 14% sucrose. The consumer test lasted four days and fresh samples were made 1 day before testing each day. Ten milliliters of each solution was measured into a 1 oz soufflé cup and stored in the refrigerator (4 °C) prior to serving.

#### 2.4.2. Testing Procedure

This study was approved by the University Institutional Review Board of the Michigan State University (East Lansing, MI) (Study ID: STUDY00004019). SIMS 2000 software (SIMS Sensory Software, Morristown, NJ, USA) was used to create and administer the questionnaire.

Consumers were instructed to rate the sweetness and bitterness intensities of the solutions on a 15-cm line scale three times, which were while the solution was in the mouth, 5 s after expectorating it, and 1 min after expectorating it. Consumers were asked to pinch their nose while holding the solution in the mouth to focus on the taste. The sweet and bitter tastes perceived at this time would be called in-mouth sweetness and bitterness throughout this paper. The perceived intensities of sweet and bitter tastes 5 s after expectorating would be referred to as immediate sweetness and bitterness. A check-all-that-apply (CATA) question on the taste was followed after evaluating the immediate tastes, including terms collected from an open-ended question in the two preliminary tests (*n*_1_ = 31 and *n*_2_ = 65), asking if the consumers noticed any aftertaste. The term *pleasant* was added to the list as a positive word, *and spicy* was added as an attention check to identify careless respondents and would be removed from the correspondence analysis. The final list of CATA consisted of 15 terms, which were *artificial, bitter, chemical, honey, licorice, metallic, minty, pleasant, pungent, spicy, sweet, tangy, tart, tingling,* and *vanilla*, and the terms were listed in alphabetical order. A 45 s break was enforced after the CATA question, which was before evaluating the sweet and bitter tastes 1 min after expectorating. The perceived intensities would be considered as lingering sweetness and bitterness. Water and crackers were provided as palate cleansers in between samples.

### 2.5. Statistical Analyses

Data analysis was performed using XLSTAT (AddinSoft, New York, NY, USA). Intensity data were analyzed using a one-factor ANOVA model. For CATA analysis, the frequencies of each attribute were counted. Cochran’s Q test was performed for each attribute to compare the difference among samples. Multiple pairwise comparisons using critical difference (Sheskin) were performed when the attribute was significant (*p* < 0.05). Correspondence analysis (CA) was generated to visually show the relationship between sensory attributes and samples. A two-way ANOVA model was used to determine the effect of PROP taster status, sweetener, and their interaction. Fisher’s least significant difference (LSD) post hoc test was performed when *p* < 0.05. Agglomerative hierarchical clustering (AHC) was used as a second way to classify PROP groups. Pearson correlation test was performed and correlation coefficients were calculated between PROP bitterness and sweet and bitter tastes of Reb A, D, and M combined over time (in-mouth, immediate, lingering sweetness and bitterness).

## 3. Results

### 3.1. Participant Characteristics

A total of 126 naïve consumers completed the study, with an average age of 23 ± 1.7 years and an average BMI of 24.7 ± 4.6 kg/m^2^ based on self-reported height and weight. None of the participants had heart disease, cancer, or diabetes. The socio-demographics of participants are shown in Table 1. The majority were female (72.2%) and 60.3% of the participants identified themselves as white. Table 2 listed out the responses of sweetener consumption behavior questions. Sucrose (81.0%) and honey (69.8%) were the most commonly consumed sweetener on a regular basis (at least once a month), followed by stevia (19.8%), sucralose (19.0%), and aspartame (19.0%), which were high-intensity sweeteners. Other sweeteners consumed (8.7%) included maple syrup, brown sugar, xylitol, high fructose corn syrup, and acesulfame K. Sixty-seven percent of participants consumed low or zero sugar added products at least once a month. More than half of the participants (54.8%) said they were somewhat or very familiar with stevia.

### 3.2. Sensory Characteristics

#### 3.2.1. Intensities of Sweet and Bitter Tastes

Table 3 summarizes the mean intensity ratings (± SEM) for four sweetener solutions evaluated by all participants. The decreasing trend in sweetness and bitterness intensities from in-mouth to immediate (5 s after expectorating the samples) to lingering (1 min after expectorating the samples) indicated that consumers followed the directions and evaluated the samples correctly, since a fading in intensity over time was expected.

The in-mouth sweetness of 14% sucrose and 0.1% Reb M were not significantly different (*p* = 0.55). The in-mouth sweetness of Reb D was slightly lower than sucrose but was still considered to be not different from sucrose (*p* = 0.19). Reb A showed significantly less in-mouth sweetness than Reb M and sucrose (*p* < 0.01 and *p* < 0.05, respectively). Reb M had the highest immediate sweetness among the samples and was significantly different from others. The sweetness of Reb M remained the highest after one minute. The lingering sweetness of Reb M (intensity = 5.3) was higher than Reb D (intensity = 4.5), but the difference was not significant (*p* = 0.05). Reb D was higher in lingering sweetness than sucrose (intensity = 3.6), but it was not significantly different (*p* = 0.05). The participants rated the in-mouth bitterness of sucrose, Reb D, and Reb M around 1, while the rating of Reb A was at 3.5 on a 15-cm line scale. The bitterness of Reb A persisted after 5 s (intensity = 3.5). Reb D was perceived to have more immediate bitterness than sucrose (*p* < 0.05), and there was no significant difference in the immediate bitterness between Reb M and sucrose (*p* = 0.27). While the lingering bitterness of sucrose, Reb D, and Reb M was at a minimum, Reb A still had detectable bitterness remaining (intensity = 1.6).

#### 3.2.2. CATA

Table 4 summarizes the total counts of CATA attributes selected by the consumer panel (*n* = 126) to describe the aftertaste of each sweetener solution. The term *sweet* was the most frequently used term, and *spicy* was the least, which were as expected. Significant differences among samples were found in 10 out of 15 attributes (*p* < 0.05). Reb A, D, and M were described as *artificial* more frequent than sucrose. The *bitter* and *chemical* tastes of Reb A were significantly higher than other sweeteners, and fewer participants considered Reb A as *sweet* and *pleasant*. *Honey* and *vanilla* were checked the most for sucrose, followed by Reb D and M, while Reb A was rarely associated with these two terms. *Licorice*, *metallic*, *minty*, *pungent*, *spicy*, *tangy*, *tart*, and *tingling* were rarely selected by participants, with no more than 15 counts for each sample. Among those 8 less-checked terms, *licorice*, *pungent*, *spicy*, *tangy*, and *tingling* were not significantly different among samples.

**Figure 1:**
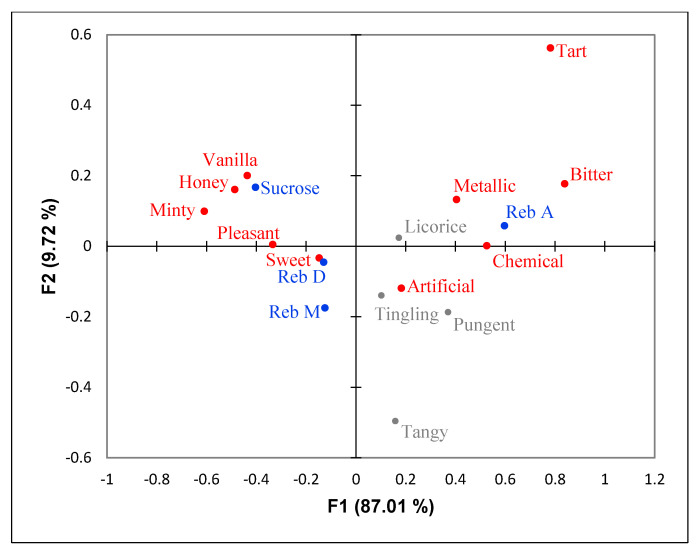
Correspondence analysis (CA) of sweeteners. Gray color indicates non-significant attributes; Red color indicates significant attributes; Samples are in blue.

The sensory attributes of sweeteners were summarized visually in Figure 1. The first two dimensions explained 96% of the variation. *Honey* and *vanilla* were associated with sucrose. Reb A was close to *metallic, bitter,* and *chemical*. Reb D and M were similar to each other and were closer to sucrose as compared to Reb A. Reb D and M were mostly associated with the positive words, but *artificial* was between Reb A and Reb D and M.

### 3.3. PROP Bitterness

#### 3.3.1. PROP Taster Groups

**Figure 2:**
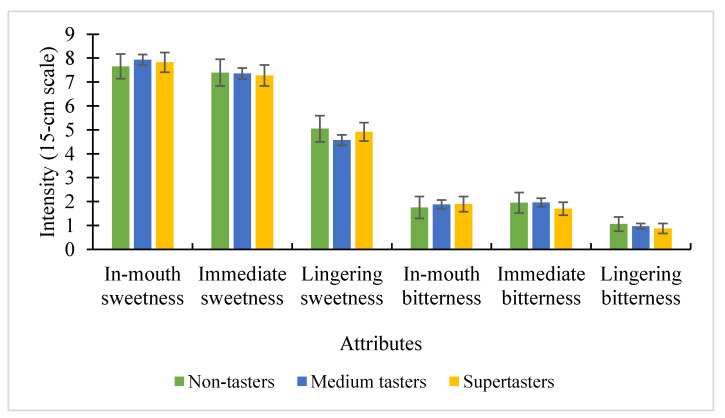
The influence of 6-n-Propylthiouracil (PROP) taster groups on the perceived intensities (± SEM), with sweeteners combined (Reb A, D, and M).

Out of 126 participants who completed the consumer test, there were 15 non-tasters, 81 medium tasters, and 30 supertasters. The interaction between taster groups and samples was not significant for all taste evaluations of each Reb A, D, and M solutions. There was also no significant difference when examining the main effect of taster groups on perceived intensity scores of sweet and bitter tastes of the three sweeteners combined over time (in-mouth, immediate, and lingering) (Figure 2).

Due to the disproportional ratio of people in each taster groups, agglomerative hierarchical clustering (AHC) was used to group people based on their dissimilarity on the PROP rating (data not shown). Three groups were generated with 55, 44, and 30 people, corresponding to low, medium, and high-sensitive clusters, respectively. However, no significant difference in the main effect of clusters was found.

#### 3.3.2. Relationships with Perceived Intensities of Reb A, D, and M

Pearson correlation tests were conducted to determine the association between PROP bitterness and perceived intensities of sweet and bitter tastes of Reb A, D, and M over time (in-mouth, immediate, lingering). No significant relationships existed between PROP bitterness and the rated intensities of the three steviol glycosides (*p* > 0.05).

## 4. Discussion

The present study investigated the sweetness and bitterness of Reb A, D, and M compared to sucrose at a high sucrose equivalent level (14% *w*/*v*) using consumers. To compare with 14% (*w*/*v*) sucrose solution, the solution concentration of the three steviol glycosides was determined by two small scale consumer tests as a preliminary test (see 2.4.1 for details). Briefly, the sweetness of 0.1% (*w*/*v*) Reb M was proved to be not significantly different from a 14% sucrose solution, and the same concentration was used for Reb A and D to compare the sensory characteristics of the three steviol glycosides at the same concentration. Prakash et al. [16] estimated that Reb M was about 200–350 times sweeter than sucrose, and the sweet potency at 10% SE was calculated to be 159. In the present study, the sweet potency of Reb M at 14% SE was calculated as 140. This is in line with the model from Prakash et al. [16], sweet potency of high-intensity sweeteners tended to decrease as the sucrose sweetness equivalent level increased [45].

The three steviol glycosides showed significant differences in sweetness and bitterness at the same concentration (0.1% *w*/*v*). The in-mouth sweetness of Reb D and M at 0.1% were not statistically different from sucrose at 14%, while 0.1% Reb A was less sweet than sucrose. Reb A was significantly less sweet than Reb M as well (*p* < 0.01) but was not significantly different from the in-mouth sweetness of Reb D (*p* = 0.24) with a tendency of being less sweet. Reb D and M were not significantly different in in-mouth sweetness (*p* = 0.06), but there was also a clear tendency of Reb D to be less sweet than Reb M. These results were consistent with the previous studies investigating the sweetness of Reb A, D, and/or M at different concentrations showing that Reb M was the sweetest sweetener and Reb A was the least sweet sweetener among Reb A, D, and M at the same concentration [15,16,46].

The sweetness temporal profile of Reb M was studied by Prakash et al. [16], who compared the sweetness appearance time and extinction time to examine the change in perception over 3 min. The sweetness of Reb M elicited later and persisted longer than sucrose at 10% SE in water. The descriptive panel rated the lingering sweetness of Reb M higher than that of sucrose as well [16]. In the present study, Reb M had a similar in-mouth sweetness to sucrose, but the lingering sweetness was significantly higher than that of sucrose, which corresponded with Prakash’s finding. Reb A was also found to have a longer extinction time than sucrose [13] and exhibited persistent flavor duration in the mouth [8]. When at a similar sweetness level (i.e., at 8% SE), the lingering sweetness of Reb A and Reb M were not different [16]. Even though, in this study, there was no significant difference in lingering sweetness between sucrose and Reb A (*p* = 0.12), the lingering sweetness of Reb A became higher than sucrose after being rated less sweet in-mouth, which suggested that if the sweetness of Reb A was at the same level as sucrose, the lingering sweetness might be significantly higher than sucrose. Reb D, like Reb M, also had a similar in-mouth sweetness to sucrose, but had marginally higher lingering sweetness than sucrose (*p* = 0.05). When comparing Reb D to Reb M, the lingering sweetness of Reb D was marginally less than that of Reb M (*p* = 0.05) similar to the in-mouth sweetness of Reb D that was almost significantly less than Reb M (*p* = 0.06). Although the lingering sweetness of Reb M seemed to be stronger than Reb D, it may due to its higher initial sweetness than Reb D.

The bitterness of Reb A stood out among the samples when consumers first tasted the sample and the bitterness continued to be significantly different from others even after one minute. Many other researchers have reported the bitterness of Reb A [13,15]. On the other hand, Reb D and M did not show much in-mouth bitterness and had a similar intensity to sucrose. Even though Reb D exhibited a significantly higher immediate bitterness than sucrose, it was still considered low. A trained panel did not detect any significant bitter taste of Reb M when comparing to sucrose at 10% SE [16]. Hellfritsch et al. [15] and Ko et al. [47] indicated that Reb D elicited a lot less bitterness than Reb A. Our results confirmed that naïve consumers like trained assessors did not detect much bitterness from Reb D and M.

Based on the total counts of CATA and the CA, Reb A was associated with some negative perception terms, such as *bitter*, *chemical*, and *artificial*. The *bitter* and *chemical* attributes were significantly more selected for Reb A than Reb D, Reb M, and sucrose. The *bitter* attribute was in agreement with the bitterness intensity rating. The bitterness and chemical sensation of Reb A was reported by Fujimaru et al. [48] as well. Significantly less *sweet* was checked for Reb A than the other sweeteners, suggesting that the bitterness and chemical sensation might overshadow the sweetness of it. Reb D and M appeared to have good taste profiles because they were close to positive terms and sucrose. However, even though Reb D and M had low citations for *bitter* and higher citations for *pleasant* than Reb A, many participants still checked *artificial* significantly more frequent than sucrose. Waldrop and Ross [49] reported that consumers did not like stevia because of its association with *artificial* flavor. Thus, the *artificial* attribute may cause negative consumer perception of Reb D and M even though they are natural sweeteners without the aversive bitter aftertaste. Interestingly, the *artificial* attribute was also selected for sucrose by 38 participants. It is not common to drink pure sugar water in daily life, so the participants may not be familiar with the taste of sucrose solutions, and thus might select *artificial* for sucrose solution.

*Licorice*, *pungent*, *spicy*, *tangy*, and *tingling* were rarely selected by the participants and were not significant to discriminate the samples. Thus, these five terms may not be appropriate terms for consumers to describe the three steviol glycosides, even though *licorice* has been commonly used to describe the aftertaste of Reb A by researchers [10,13,50] and media [18]. The *licorice* taste of Reb A did not exhibit at low SE levels, but was elicited at higher SE levels [13], and this was further proved by Reyes et al. [50] that Reb A at 0.1% had more notable *licorice* taste than at 0.012%. In this study, we did not find the correlation between *licorice* and Reb A at 0.1%, since only 8 people out of 126 selected it, which suggested that *licorice* may not be an appropriate term for consumers to describe the aftertaste of Reb A or to discriminate Reb A, D, and M.

The CATA analysis also found that *vanilla* and *honey* were associated with sucrose. A consumer survey showed that honey was the most popular sugar alternative, which was natural, and natural sweeteners were perceived better than artificial sweeteners in general [3]. In this study, those who checked *honey* for steviol glycosides might imply that the sample gave them a sense of natural. As for *vanilla*, Lavin and Lawless [51] showed that an added vanilla flavor enhanced the perception of sweetness in milk, and Wang et al. [52] also indicated a taste-aroma interaction between perceived sweetness and vanilla flavor in skim milk. Vanilla was congruent with sweetness, so participants might choose the term even though the attribute was not presented in the solution.

A secondary objective of this study was to investigate the influence of consumers’ PROP taster status on the sweetness and bitterness of Reb A, D, and M. We found that there were no significant differences in the perceived sweetness and bitterness of Reb A, D, and M (in-mouth, immediate, and lingering) among PROP taster groups. Risso et al. [40] reported that there was no correlation between PROP bitterness and stevioside bitterness. Humans have about 25 bitter taste receptors from the taste 2 receptors (hTAS2Rs) gene family [53]. Each receptor responds to different compounds but may have overlapped molecular range [54]. The sensitivity to the bitterness of PROP/PTC is mainly associated with TAS2R38 bitter taste receptor [55,56]. TAS2R4 and TAS2R14 responded to the bitterness of stevioside and Reb A in vitro, while TAS2R38 did not react [15]. Meyerhof et al. [54] sorted receptors into 4 groups, and both TAR2S4 and TAS2R14 were not in the same group as TAS2R38. The different responses in bitter taste receptors might explain why no relationship was found between PROP taster status and perceived bitterness intensity of Reb A, D, and M.

Some studies suggested that PROP bitterness sensitivity influenced other oral sensations, such as sweetness [36,37,57,58,59,60,61]. Drewnowski et al. [36] found a weak and marginal significant difference in sweetness perception of sucrose and saccharin between PROP tasters and non-tasters, and the difference was more significant at lower concentrations. Allen et al. [59] reported that the sweetness of acesulfame potassium was positively associated with PROP bitterness. A large sample size study (*n* > 1500) found a weak association between sweetness and PROP bitterness, suggesting that a bigger size sample is required to detect weak association with PROP [60]. A recent study confirmed that PROP bitterness was positively correlated with sweetness of sucrose [61]. However, some of the previous studies also indicated that there was no relationship between PROP sensitivity and sweet taste responsiveness [62,63,64]. Here, we found no significant differences in perceived sweetness intensity among PROP groups and further, no correlation between PROP bitterness and sweetness of the steviol glycosides. In this study, the test stimuli were singles (i.e., each sweetener solution), but the aftertaste of the three steviol glycosides, especially the sweet-bitter Reb A at a high concentration, might cause difficulties for participants to evaluate intensities of sweet and bitter tastes. Horne et al. [38] reported that sweet-bitter stimuli might be more difficult to evaluate than single taste stimuli due to taste–taste interactions. Expansive, linear, and compressive phases of psychophysical functions could be used to predict how taste stimuli would behave when mixed at low, medium, and high intensity/concentration [65]. For example, perceptual enhancement and suppression has been extensively reported at low and high intensity/concentration mixtures, corresponding to the expansive phase and compressive phase of the psychophysical function, respectively [65]. Ly and Drewnowski [63] showed a reduced difference in bitterness between PROP taster groups was found when the caffeine solution was sweetened, even though PROP tasters rated caffeine solution without sweetener as more bitter than non-tasters [63]. The perceptual suppression as a result of sweet-bitter interaction at a high intensity/concentration may explain no differences in perceived sweetness intensity among PROP groups and no correlations between PROP bitterness and perceived sweetness of Reb A, D, and M.

One possible limitation of the study was that consumers did not swallow the solution, which limited the number of taste buds utilized for the evaluation. Taste buds are distributed not only in the mouth but also in the throat [66]. Consumers were asked to expectorate the sample to reduce fatigue, however, the swallowing sensation could be different and might impact the perceived intensities. No hedonic question was asked in this study because it might be difficult for naïve consumers to rate the likings of pure sweetener solutions when the solutions were not regularly consumed in daily life. However, no association could be drawn between the negative CATA attributes and the likings of the sweeteners. Another limitation was the disproportional size of PROP groups, which only had 15 non-tasters. The data from non-tasters might be less variable if more non-tasters were recruited.

## 5. Conclusions

The present study investigated the sensory profile of Reb A, D, and M at 14% SE using a consumer panel, and the influence of PROP taster groups on the perceived sweet and bitter tastes of the three glycosides. Reb D and M had sensory profiles that were closer to sucrose, compared to Reb A, but were still associated with negative sensation, such as *artificial*, which may cause negative perception toward Reb D and M. The lingering sweetness of Reb D and M was also a concern. The sensory characteristics of Reb A, D, and M in this study can be used as a reference for the food industries working with steviol glycosides in high-sugar applications, such as frozen desserts. Furthermore, there were no significant differences among non-tasters, medium tasters, and supertasters on the perceived sweetness and bitterness of Reb A, D, and M as well as no significant correlations between PROP bitterness and perceived sweet and bitter tastes, suggesting that supertasters who experience more intense taste sensations may not report aversive sensations from stevia. Further studies on the consumer acceptance of Reb A, D, and M are needed to determine if these characteristics would affect the likings of these sweeteners. Since the sweeteners may perform differently in a food matrix than in aqueous solution, more research using steviol glycosides in final food products are needed to determine the sensory profile and acceptance of them.

## Figures and Tables

**Table 1:** Socio-demographic characteristics of participants (*n* = 126).

Variable	Definition	Frequency	%
Gender			
	Male	35	27.8%
	Female	91	72.2%
Ethnicity			
	White	76	60.3%
	Hispanic or Latino	5	4.0%
	Asian or Pacific Islander	33	26.2%
	Black or African American	7	5.6%
	Native American or American Indian	0	0.0%
	Other	3	2.4%
	Prefer not to respond	2	1.6%
Education			
	Less than high school	0	0.0%
	High school diploma or GED	29	23.0%
	2-year college degree	4	3.2%
	4-year college degree	50	39.7%
	Graduate degree (Master’s, Doctorate, etc.)	43	34.1%

**Table 2:** Participants’ sweetener consumption behavior (*n* = 126).

Characteristic	Definition	Frequency	%
Sweetener consumption ^1^			
	Agave nectar	16	12.7%
	Aspartame	24	19.0%
	Erythritol	10	7.9%
	Honey	88	69.8%
	Monk fruit extract	9	7.1%
	Saccharin	12	9.5%
	Stevia	25	19.8%
	Sucralose	24	19.0%
	Sucrose	102	81.0%
	Others	11	8.7%
Low/zero sugar added product consumption frequency			
	More than 3 times a week	16	12.7%
	1–2 times a week	29	23.0%
	2–3 times a month	27	21.4%
	Once a month	12	9.5%
	Every other month	6	4.8%
	1–2 times per 6 months	15	11.9%
	Less than once a year	7	5.6%
	Almost never	14	11.1%
Familiarity with stevia			
	Very unfamiliar	25	19.8%
	Somewhat unfamiliar	21	16.7%
	Neutral	11	8.7%
	Somewhat familiar	55	43.7%
	Very familiar	14	11.1%

^1^ This is a check-all-that-apply question.

**Table 3:** Mean intensity scores (± SEM) of sweetener solutions by participants (*n* = 126).

Sweetener	Sweetness ^1^			Bitterness ^2^		
	In-Mouth	Immediate	Lingering	In-Mouth	Immediate	Lingering
Sucrose	8.3 ± 0.3 ^3^ a ^4^	7.1 ± 0.3 b	3.6 ± 0.3 b	0.8 ± 0.1 b	0.6 ± 0.1c	0.4 ± 0.1b
Reb A	7.2 ± 0.3 b	6.5 ± 0.3 b	4.3 ± 0.3 b	3.5 ± 0.3 a	3.5 ± 0.3 a	1.6 ± 0.2 a
Reb D	7.8 ± 0.3 ab	7.2 ± 0.3 b	4.5 ± 0.3 ab	1.1 ± 0.2 b	1.3 ± 0.2 b	0.6 ± 0.1 b
Reb M	8.6 ± 0.3 a	8.2 ± 0.3 a	5.3 ± 0.3 a	1.0 ± 0.2 b	0.9 ± 0.1 bc	0.6 ± 0.1 b

^1,2^ In-mouth tastes (sweetness and bitterness) were evaluated when the solution was in the mouth; Immediate tastes were evaluated 5 s after expectorating the sample; Lingering tastes were evaluated 1 min after expectorating the sample. ^3^ Intensities were evaluated on a marked 15-cm line scale anchored with “not at all” to “extremely”. ^4^ Different letters in the same column show the significant differences between sample means at *p* < 0.05 by Fisher’s LSD.

**Table 4:** Total counts of check-all-that-apply attributes for sweetener solutions.

Attribute	Sucrose	Reb A	Reb D	Reb M
Artificial ***	38 b	83 a	64 a	69 a
Bitter ***	8 b	66 a	17 b	12 b
Chemical ***	9 b	42 a	17 b	18 b
Honey ***	41 a	8 c	25 b	24 b
Licorice ^ns^	5	8	4	6
Metallic *	6 a	15 a	6 a	6 a
Minty **	7 ab	0 b	9 a	3 ab
Pleasant ***	65 a	25 b	49 a	57 a
Pungent ^ns^	2	7	2	6
Spicy ^ns^	0	0	2	1
Sweet***	110 a	83 b	110 a	114 a
Tangy ^ns^	1	5	4	8
Tart *	2 ab	6 a	1 ab	0 b
Tingling ^ns^	4	7	6	7
Vanilla ***	27 a	7 b	15 ab	15 ab

* indicates *p* < 0.05, ** indicates *p* < 0.01, *** indicates *p* < 0.001, and ns indicates no significant differences among samples. Different letters in the same row indicate the significant differences between sample means at *p* < 0.05 by Critical Difference (Sheskin).
